# Inverse Association between Cheese Consumption and Lower Cognitive Function in Japanese Community-Dwelling Older Adults Based on a Cross-Sectional Study

**DOI:** 10.3390/nu15143181

**Published:** 2023-07-18

**Authors:** Hunkyung Kim, Yosuke Osuka, Narumi Kojima, Hiroyuki Sasai, Kentaro Nakamura, Chisato Oba, Mayuki Sasaki, Takao Suzuki

**Affiliations:** 1 Gaon Research Center, 402 Pangyo Medical Tower, Seongnam-shi 13466, Republic of Korea; 2 Department of Frailty Research, Center for Gerontology and Social Science, Research Institute, National Center for Geriatrics and Gerontology, Obu 474-8511, Aichi, Japan; osuka@ncgg.go.jp; 3 Research Team for Promoting Independence and Mental Health, Tokyo Metropolitan Institute of Gerontology, Itabashi 173-0015, Tokyo, Japan; nkojima@tmig.or.jp (N.K.); sasai@tmig.or.jp (H.S.); 4 Nutrition and Food Function Research Department, R&D Division, Meiji Co. Ltd., Hachioji 192-0919, Tokyo, Japan; kentarou.nakamura@meiji.com (K.N.); chisato.ohba@meiji.com (C.O.); mayuki.sasaki@meiji.com (M.S.); 5 Institute of Gerontology, J. F. Oberlin University, Machida 194-0294, Tokyo, Japan; suzutaka5651@gmail.com

**Keywords:** cheese, MMSE score, lower cognitive function

## Abstract

Diet modification may contribute to the prevention of age-related cognitive decline. The association between dairy product consumption and cognitive function in older people remains unknown. We investigated whether cheese intake is associated with lower cognitive function (LCF) in community-dwelling older adults. This cross-sectional study included 1503 adults aged over 65 years. The analyzed data were obtained through face-to-face interviews and functional ability measurement. Cognitive function was assessed using the mini-mental state examination (MMSE), and a score ≤23 was defined as LCF. The prevalence of LCF was 4.6%, and this group had smaller calf circumference, slower usual walking speed, and a more frequent history of anemia than subjects with MMSE scores >23. After adjusting for confounding factors, logistic regression analysis revealed cheese intake (odds ratio (OR) = 0.404, 95% confidence interval (CI) = 0.198–0.824), age (OR = 1.170, 95% CI = 1.089–1.256), usual walking speed (OR = 0.171, 95% CI = 0.062–0.472) and calf circumference (OR = 0.823, 95% CI = 0.747–0.908) to be significant factors associated with LCF. Although the present study was an analysis of cross-sectional data of Japanese community-dwelling older adults, the results suggest that cheese intake is inversely associated with LCF.

## 1. Introduction

Dementia prevention and treatment are major strategies for achieving good health and longevity. The proportion of the older population with dementia, and the percentage of people requiring nursing care due to dementia, continue to increase; as such, dementia prevention has become an urgent issue [1,2].

The effective prevention of dementia requires preserving as much cognitive function as possible and preventing cognitive decline as early as possible. A variety of information obtained from longitudinal and cross-sectional research provides insight into the factors that affect cognitive function. A synopsis of the results of previous studies has indicated that physical activity, consumption of a Mediterranean diet, dairy intake in midlife, and moderate consumption of wine are effective in delaying or preventing dementia and cognitive decline [3,4,5,6]. On the other hand, a history of diabetes, vitamin K or vitamin D deficiency, elevated D-type amino acids, and frailty are known factors that promote dementia and cognitive decline [7,8,9,10,11].

In particular, there is increased interest in the relationship between food intake and cognitive function. Previous studies have shown that a dietary pattern characterized by a high intake of soybean products, vegetables, seaweed, milk, and dairy products, together with a low intake of grain products, is associated with reduced risk of developing dementia [12]; moreover, a high intake of milk and dairy products reduces the risk of developing dementia, especially Alzheimer’s dementia [13]. Recently, it has been found that a high intake of dark green vegetables, rich in phylloquinone, beta-carotene, and alpha-tocopherol, could benefit certain domains of cognition in older adults, such as learning and memory [14]. De Goeij et al. [15] reported that cheese intake was associated with information processing speed but not with memory and suggested that the influence may differ depending on the cognitive function subscale. Suzuki et al. [16] conducted a randomized controlled study in which older women with mild cognitive impairment consumed mature cheese for 3 months, and they reported a significant increase in brain-derived neurotrophic factor but no significant change in mini-mental state examination (MMSE) score [17]. Ni et al. [18] analyzed a 2-year follow-up data on the association between dairy product intake and changes in cognitive function and found no association between low-fat milk, yogurt, cheese, and fermented dairy intake with changes in cognitive performance. The results of analyses of the association between dairy product intake and cognitive function obtained via observational and/or intervention studies differ among studies. Moreover, Lee et al. [19] concluded that the existing evidence is not sufficient to conclude that milk and dairy product intake contributes to a reduced risk of cognitive decline. Further studies are needed to determine the role of dairy intake in cognitive function in older adults.

The purpose of this study was to elucidate the relationship between cheese intake and cognitive function, evaluated based on MMSE scores in community-dwelling older people, using cross-sectional data.

## 2. Materials and Methods

### 2.1. Subjects

This study was conducted as part of a comprehensive geriatric survey that aimed to identify factors related to geriatric syndromes, such as cognitive impairment, falls, urinary incontinence, frailty, and sarcopenia. Participants were recruited from the participant pool of a comprehensive geriatric survey conducted by our team once per year or every two years. Participants were included in this study if (i) they were community-dwelling older adults aged 65–99 years and (ii) they had provided written informed consent. Participants were excluded from the analyses if (i) they did not consent to their data being used; (ii) they had an unstable medical condition, a severe disease, or were not permitted to participate in this study by a study physician, or (iii) had participated in other cohort studies.

This study analyzed the cross-sectional data of two different cohorts. Cohort 1 consisted of 759 people from a previous 2017 cohort (see below for details) who had participated in the follow-up survey in 2019. The data of this cohort from the 2019 survey were used as the cross-sectional data for this study. Cohort 2 consisted of 757 people who had participated in a new cohort survey in 2019. The total number of subjects was 1516. The recruitment processes for the two cohorts are described below.

*Cohort 1*: This cross-sectional study utilized data from a cohort of subjects who had participated in a comprehensive health examination, “The Otassha Study 2017 Cohort”, conducted at the Tokyo Metropolitan Institute of Gerontology [20]. Older women aged 65 years or older living in Itabashi, Tokyo, Japan, were recruited using the Basic Resident Register (*n* = 6788) in 2017. After excluding 422 women who had participated in another cohort study, we sent invitation letters to 6366 candidates. A total of 1365 women participated in the health examination in 2017. Of these subjects, 759 women (55.6%) completed the follow-up examination in 2019 (Baseline survey in 2017, and every two years follow-up survey for a period of 10 years).

*Cohort 2:* This study recruited volunteer participants from 18 neighborhoods near the Tokyo Metropolitan Institute of Gerontology, Itabashi, Tokyo, Japan. In 2019, the names and addresses of all individuals aged 75–85 years and registered in the Basic Resident Registry for this area were extracted (*n* = 4233). After excluding 88 participants who had been enrolled in other cohorts, invitations were sent to 4145 candidates. In total, 757 individuals (275 men and 482 women) participated in this study (Baseline survey in 2019 and once-per-year follow-up survey for a period of 10 years).

Out of 1516 subjects in two cohorts, 1504 subjects with no missing values for cheese intake and MMSE were analyzed.

The study protocol was approved by the Clinical Research Ethics Committee of the Tokyo Metropolitan Institute of Gerontology (TMIG) (ID R2-25 and R2-18). The procedures were fully explained to all participants, and written informed consent was obtained.

### 2.2. Outcome Measures

*Interview*. Face-to-face interviews were conducted to assess the participants’ history of falls, urinary incontinence, and frequencies of food intake (cheese, milk, fish, meat, egg, soy products, potatoes, fruit, seaweed, green and yellow vegetables, and fats and oils), and chronic conditions, such as heart disease, hyperlipidemia, dyslipidemia, diabetes, osteoporosis, osteoarthritis (OA), and anemia. The dietary variety score was calculated using the intake frequencies of 9 food categories (fish, meat, eggs, soy products, potatoes, fruit, seaweed, green and yellow vegetables, and fats and oils; in the original paper, 10 items were calculated, including milk, but in this study, milk was considered to be in the same family as cheese, so it was excluded). For each participant, a score of 1 was given for each food category if it was eaten every day, and a score of 0 was given if it was eaten once every 2 days, once or twice a week, or not at all; the total score (0 to 9) was then calculated [21].

*Anthropometric and physical function measures*. Measurements of height and weight were converted to body mass index (BMI). Muscle mass, body fat mass, and percentage of body fat were determined using bioelectrical impedance analysis (BIA) (InBody 720, Biospace, Seoul, Republic of Korea). Blood pressure was measured using automatic blood pressure monitors (TM-2657, A&D, Japan) prior to the measurement of physical function. The calf circumference of the non-dominant leg was measured with the subject in a seated position, with the knee and ankle at right angles and the foot resting on the floor. Measurements were taken at the level of the widest circumference, and subcutaneous tissue was not compressed. The grip strength of the dominant hand was measured twice using a hand-held Smedley-type dynamometer, and the highest value of the two trials was used for analysis. Usual walking speed was measured twice on a flat walking path of 11 m with markers placed at 3 m and 8 m. A stopwatch was used to measure the time taken to walk 5 m between the markers, and the fastest time of the two trials was recorded. The use of assistive walking devices was allowed in the measurement of walking speed if the participant expressed concern about walking without a device or if the investigators suspected a risk of falling.

*Blood indicators*. Non-fasting blood samples were collected in a seated position at baseline. Analyses were carried out centrally in one laboratory (Special Reference Laboratories, Tokyo, Japan). Lipid levels (total cholesterol, high-density lipoprotein (HDL) cholesterol, and triglycerides) were determined. Serum albumin was measured via the bromocresol green method, glycated hemoglobin (HbA1c) via latex agglutination assay, and serum creatinine via an enzymatic assay.

*Cognitive function*. Global cognitive status was assessed using MMSE. Lower cognitive function was operationally defined as an MMSE score ≤23 [22].

### 2.3. Data Analysis

Descriptive statistics are expressed as mean and standard deviation or as a number (%). Participants were divided into a cheese intake group (N = 1230) and a non-cheese intake group (N = 287).

The subjects were also classified into a group with MMSE scores ≤ 23 and a group with MMSE scores > 23. The two groups were compared using Student’s *t*-test for continuous variables and using a chi-square test for categorical variables. Multiple logistic regression analyses were used to analyze the factors associated with lower cognitive function. Model I included only the participants’ cheese intake. Model II was Model I adjusted for age, physical function, and physique factors. Model III was Model II adjusted for medical history, blood pressure, number of teeth, blood variables, urinary incontinence, milk intake, and dietary variety score. Significant and nonsignificant variables were entered into the multiple logistic regression models to obtain the odds ratio (OR) and 95% confidence interval (CI).

The *p*-values of less than 0.05 were considered statistically significant. All analyses were performed using SPSS software, Windows version 25.0 (SPSS Inc., Tokyo, Japan).

## 3. Results

Table 1 shows the cheese intake of the subjects. Of a total of 1517 subjects, 1230 (81.0%) comprised the cheese intake group, and 287 (19.0%) comprised the non-cheese intake group. Regarding the type of cheese consumed, processed cheese accounted for the highest percentage of 65.7%, and white mold cheese accounted for 15.3%.

Table 2 shows a comparison of the measured parameters between the cheese intake group and the non-cheese intake group. Compared to the cheese intake group, the non-cheese group had a slower usual walking speed, fewer teeth, lower total cholesterol and HDL cholesterol levels, a higher prevalence of urinary incontinence, a lower percentage of milk consumption, and a lower total MMSE score.

**Figure 1:**
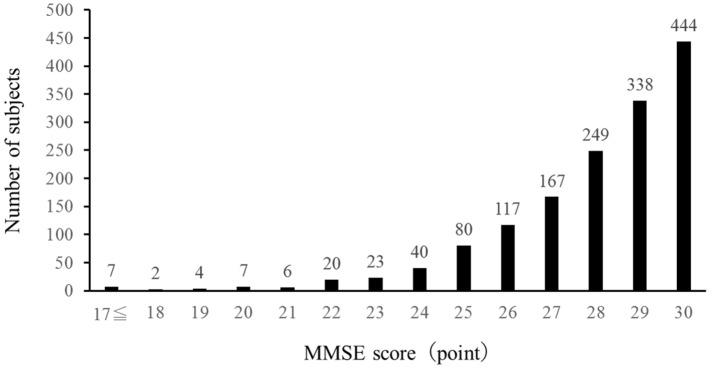
Frequency of MMSE scores.

Figure 1 shows the distribution of the changes in the MMSE scores among subjects. Sixty-nine subjects had MMSE scores of 23 or below.

Table 3 shows a comparison of the measured parameters between the MMSE > 23 group and the MMSE ≤ 23 group. Compared to the MMSE > 23 group, the MMSE ≤ 23 group were older and had smaller calf circumference, slower usual walking speed, lower total MMSE score, fewer teeth, higher creatinine levels, a lower total cholesterol and HDL cholesterol levels, lower albumin levels, a higher prevalence of anemia, and a lower percentage of milk consumption.

As shown in Table 4, multiple logistic regression (model III) identified four variables, i.e., cheese intake (OR = 0.404, 95% CI = 0.198–0.824), age (OR = 1.170, 95% CI = 1.089–1.256), usual walking speed (OR = 0.171, 95% CI = 0.062–0.472), and calf circumference (OR = 0.823, 95% CI = 0.747–0.908) as significant independent variables for lower cognitive function. In all three models, cheese intake was identified as a factor that was significantly inversely associated with lower cognitive function.

## 4. Discussion

In the present study, we conducted an analysis of cross-sectional data on the relationship between cheese intake and cognitive function in community-dwelling older people, and the results suggest that cheese intake is inversely associated with the risk of lower cognitive function, defined as an MMSE score of 23 or below, even after adjusting for multiple confounding factors. The significance of the results of this research will be discussed from various points of view.

Many studies on the relationship between food intake and the development of cognitive decline and dementia have been reported in multiple research fields. Among 1081 people aged 60 and above without dementia at baseline, who were assessed for 17 years (1988–2005) in the Hisayama study, 303 people subsequently developed dementia (including 166 patients with Alzheimer’s dementia and 98 patients with vascular dementia). When the relationship between milk and dairy product intake and dementia was analyzed in this cohort, it was found that milk and dairy product intake significantly reduced the risk of developing Alzheimer’s dementia (*p* = 0.03) but not vascular dementia (*p* = 0.14), even after adjusting for many potential confounders including age, sex, years of education, stroke, hypertension, diabetes, total cholesterol level, BMI, smoking, and exercise [13]. Another study using a 3-day weighed food record found that in women, an increased intake of grain products increased their risk of cognitive decline by 40%, whereas an increased intake of dairy products reduced the risk of cognitive decline by 20% [12]. However, according to a meta-analysis, the existing evidence, which is derived mostly from observational studies, is not sufficient to arrive at a definitive conclusion regarding the effect of milk or dairy product intake on the risk of cognitive decline or cognitive disorders in older adults [19]. The present study also detected no association between the frequency of milk consumption and MMSE scores of 23 or less, which is consistent with the conclusion of Lee et al. [19]. On the other hand, cheese intake was found to be a factor that was inversely associated with lower cognitive function. A previous study using logistic regression analyses showed that a 30 g increase in Dutch cheese intake was associated with a 33% lower probability of poor information processing speed (PR = 0.67, 95% CI: 0.47–0.97), whereas dairy intake was not associated with attention and working memory or episodic memory [15]. Another study reported that cheese intake was inversely associated with cognitive impairment in a simple logistic regression analysis (OR = 0.59; 95% CI: 0.42, 0.84; *p* = 0.003) and a multiple logistic regression analysis (OR = 0.68; 95% CI: 0.47, 0.99; *p* = 0.04) after adjusting for socio-demographic factors and other dietary factors, leading to the conclusion that increased cheese intake was associated with decreased cognitive impairment (*p* = 0.0034) [23]. The outcomes of a cohort study revealed that higher cheese intake was associated with a lower risk of incident dementia; those in the highest (>31 g/day) compared with those in the lowest (<0.7 g/day) cheese intake quartile had a 28% lower multivariable-adjusted risk of incidence [24]. A large cohort study of community-dwelling older adults showed that cheese, among other dairy products, was positively associated with the executive function domain [25].

To explore the relationship between cheese intake and cognitive function in detail, we conducted multiple regression analyses using three models. The results shown in Table 4 indicate that cheese intake was significantly inversely associated with lower cognitive function, even after adjusting for a variety of confounders, including a history of anemia and milk intake.

Although the present study shows an inverse association of cheese intake with lower cognitive function, it is not possible to elucidate the cause of this association from the results obtained from the cross-sectional data. However, a previous study reported an association between higher dietary diversity and better cognitive function [26]. In the present study, the cheese intake group had significantly higher dietary variety scores than the non-cheese intake group (Table 2). This result may suggest that the inverse association between cheese intake and lower cognitive function may be due to the likelihood that subjects with cheese intake had a dietary habit of consuming a wide variety of foods rather than the specific nutrients contained in cheese. However, multiple logistic regression analysis uncovered that dietary variety score was not distinguished as a significant independent variable for lower cognitive function (Table 4), indicating that the possibility that cheese contains specific nutrients which support cognitive function still cannot be denied. In addition to the dietary variety score, blood indicators, such as total cholesterol and HDL cholesterol levels, were lower in the non-cheese intake group compared to the cheese intake group in this study (Table 2). These results were inconsistent with the previous study that stated a lower prevalence of hypertriglyceridemia and lower HDL cholesterol plasma levels [27]. On the contrary, it was possible that dietary habits affected blood indicators. Hayakawa et al. [21] reported that blood components, such as serum creatinine and HbA1c, were significantly different among the dietary variety score groups. In fact, the results of our study also revealed that after multiple logistic regression analyses, the total cholesterol and HDL cholesterol levels were not identified as significant variables for lower cognitive function (Table 4), and this corresponds to the results from another previous study by Dalmeijer et al. [28]. Overall, further studies are required to clarify the correlation of cheese intake with cognitive function or blood indicators.

In this study, the factors, other than cheese intake, that were associated with lower cognitive function were age, usual walking speed, and calf circumference. Many previous studies have reported that age is associated with cognitive decline and dementia, and this finding was validated in this study. The association of usual walking speed with cognitive decline and lower cognitive function has been demonstrated and verified in longitudinal and cross-sectional studies [29,30], and the results of the present study were consistent with previous findings. Calf circumference is another important factor. Many previous studies have pointed out a strong association between calf circumference and frailty or sarcopenia, and the measurement of calf circumference has been adopted as a screening test for sarcopenia [31,32]. However, a relationship between calf circumference and cognitive function has not been reported. The present study also suggests the possibility that a larger calf circumference is associated with a reduced risk of lower cognitive function. Regarding the effect of calf circumference on cognitive function, the mechanism cannot be elucidated from the results of the present study.

This study has several limitations. First, the present finding of an inverse relationship between cheese intake and lower cognitive function was obtained from an analysis of cross-sectional data. Whether cheese intake contributes to a reduced risk of cognitive decline cannot be elucidated from the present result, and should be further investigated in a longitudinal study. Second, information on the status of cheese intake was based on self-reporting during the interview, and was not quantified by an objective method. Third, although many cut-off values have been proposed for defining lower cognitive function, the cut-off value used in the present study was operationally determined as an MMSE score of 23 or below, according to that proposed by O’Bryant et al. [22]. Finally, when the incidence of events is high (in cohort studies and RCTs), the OR can be highly misleading as it exaggerates the size of the effect [33].

## 5. Conclusions

Although the present study was an analysis of cross-sectional data of Japanese community-dwelling older adults, the results suggest that cheese intake is inversely associated with lower cognitive function even after adjusting for multiple confounding factors. In the future, a large-scale longitudinal analysis is needed to elucidate the causal relationship.

## Figures and Tables

**Table 1:** Cheese intake of study participants.

Variables	Category	N	%
Cheese intake	Daily	418	27.6
	Once every two days	358	23.7
	1–2 times a week	449	29.7
	No intake	287	19.0
Type of cheese *	Processed cheese	1082	65.7
	Fresh cheese	215	13.0
	White mold cheese	252	15.3
	Blue mold cheese	41	2.5
	Other	57	3.5

* Multiple answers. N: number.

**Table 2:** Comparison of selected variables between cheese intake and non-cheese intake groups.

Variable	Cheese Intake	N	M		SD	*t*-Value ^†^	*p*-Value
Age, yrs	Yes	1225	76.29	±	4.82	1.445	0.074
	No	287	76.75	±	4.78		
BMI, kg/m^2^	Yes	1213	22.70	±	3.35	0.695	0.244
	No	279	22.85	±	3.32		
SBP, mmHg	Yes	1225	134.38	±	17.46	0.741	0.229
	No	287	135.24	±	18.29		
DBP, mmHg	Yes	1225	75.64	±	11.23	0.439	0.330
	No	287	75.97	±	12.19		
% body fat, %	Yes	1212	29.48	±	8.95	0.027	0.489
	No	277	29.49	±	8.71		
Calf circumference, cm	Yes	1225	34.37	±	3.28	1.097	0.136
	No	286	34.14	±	3.08		
Grip strength, kg	Yes	1221	22.28	±	6.14	0.788	0.216
	No	286	21.93	±	6.75		
Usual walking speed, m/s	Yes	1223	1.30	±	0.25	3.517	<0.001
	No	281	1.24	±	0.26		
MMSE score	Yes	1218	28.11	±	2.16	4.412	<0.001
	No	285	27.24	±	3.17		
Number of teeth, N	Yes	1224	20.00	±	8.56	2.242	0.013
	No	287	18.66	±	9.27		
Creatinine, mg/dL	Yes	1223	0.77	±	0.34	0.788	0.215
	No	287	0.78	±	0.24		
Total cholesterol, mg/dL	Yes	1223	214.52	±	34.92	2.120	0.017
	No	287	209.67	±	34.57		
HDL cholesterol, mg/dL	Yes	1223	68.47	±	17.70	2.339	0.010
	No	287	65.77	±	17.15		
Triglycerides, mg/dL	Yes	1223	145.40	±	84.91	0.155	0.438
	No	287	146.26	±	86.60		
Albumin, g/dL	Yes	1223	4.29	±	0.27	0.395	0.346
	No	287	4.28	±	0.29		
HbA1c, %	Yes	1222	5.99	±	0.58	1.239	0.108
	No	287	5.94	±	0.60		
Number of chronic diseases, N	Yes	1221	1.90	±	1.31	0.017	0.493
	No	286	1.90	±	1.35		
Dietary variety score, point	Yes	1218	4.65	±	2.05	6.538	<0.001
	No	285	3.77	±	2.04		
Anemia: yes, %	Yes	1223	3.7			2.725	0.099
	No	287	1.7				
Urinary incontinence: yes, %	Yes	1225	37.1			4.042	0.044
	No	287	43.6				
Milk intake: yes, %	Yes	1225	79.3			57.773	<0.001
	No	287	57.8				

Data are presented as mean (M) and standard deviation (SD) for continuous variables and percentage for categorical variables. BMI: body mass index; SBP: systolic blood pressure; DBP: diastolic blood pressure; MMSE: mini-mental state examination; HDL: high-density lipoprotein; HbA1c: hemoglobin A1c; N: number. ^†^ Student’s *t*-test for categorical variables and chi-square for categorical variables.

**Table 3:** Comparison of selected variables between MMSE score ≦ 23 and >23 groups.

Variable	Group	N	M		SD	*t*-Value ^†^	*p*-Value
Age, yrs	MMSE ≦ 23	69	80.13	±	4.04	6.750	<0.001
	MMSE > 23	1435	76.18	±	4.77		
BMI, kg/m^2^	MMSE ≦ 23	67	22.37	±	3.64	0.903	0.183
	MMSE > 23	1417	22.75	±	3.32		
SBP, mmHg	MMSE ≦ 23	69	134.99	±	19.24	0.208	0.418
	MMSE > 23	1435	134.54	±	17.50		
DBP, mmHg	MMSE ≦ 23	69	75.75	±	13.99	0.019	0.491
	MMSE > 23	1435	75.72	±	11.27		
Percentage body fat, %	MMSE ≦ 23	67	30.56	±	11.02	1.064	0.144
	MMSE > 23	1414	29.38	±	8.75		
Calf circumference, cm	MMSE ≦ 23	69	32.49	±	3.56	4.424	<0.001
	MMSE > 23	1434	34.42	±	3.21		
Grip strength, kg	MMSE ≦ 23	68	20.62	±	8.29	1.661	0.051
	MMSE > 23	1431	22.31	±	6.15		
Usual walking speed, m/s	MMSE ≦ 23	68	1.10	±	0.27	6.365	<0.001
	MMSE > 23	1428	1.30	±	0.25		
MMSE score	MMSE ≦ 23	69	20.67	±	3.62	17.436	<0.001
	MMSE > 23	1435	28.30	±	1.67		
Number of teeth, N	MMSE ≦ 23	69	16.00	±	9.58	3.342	0.001
	MMSE > 23	1434	19.93	±	8.62		
Creatinine, mg/dL	MMSE ≦ 23	69	0.97	±	0.87	2.010	0.024
	MMSE > 23	1433	0.76	±	0.27		
Total cholesterol, mg/dL	MMSE ≦ 23	69	205.00	±	36.04	2.116	0.017
	MMSE > 23	1433	214.07	±	34.71		
HDL cholesterol, mg/dL	MMSE ≦ 23	69	62.87	±	16.88	2.453	0.007
	MMSE > 23	1433	68.17	±	17.57		
Triglyceride, mg/dL	MMSE ≦ 23	69	146.94	±	99.56	0.122	0.451
	MMSE > 23	1433	145.66	±	84.69		
Albumin, g/dL	MMSE ≦ 23	69	4.19	±	0.31	3.171	0.001
	MMSE > 23	1433	4.29	±	0.27		
HbA1c, %	MMSE ≦ 23	69	6.05	±	0.73	1.083	0.140
	MMSE > 23	1432	5.97	±	0.58		
Number of chronic diseases, N	MMSE ≦ 23	69	1.74	±	1.50	0.939	0.176
	MMSE > 23	1430	1.91	±	1.31		
Dietary variety score, point	MMSE ≦ 23	68	3.88	±	2.15	2.474	0.013
	MMSE > 23	1428	4.52	±	2.06		
Anemia: yes, %	MMSE ≦ 23	69	8.7			6.473	0.011
	MMSE > 23	1433	3.1				
Urinary incontinence: yes, %	MMSE ≦ 23	69	29.0			2.727	0.099
	MMSE > 23	1435	37.1				
Milk intake: yes, %	MMSE ≦ 23	69	65.2			3.922	0.048
	MMSE > 23	1435	75.7				

Data are presented as mean (M) and standard deviation (SD) for continuous variables and percentage for categorical variables. BMI: body mass index; SBP: systolic blood pressure; DBP: diastolic blood pressure; MMSE: mini-mental state examination; HDL: high-density lipoprotein; HbA1c: hemoglobin A1c; N: number. ^†^ Student’s *t*-test for categorical variables and chi-square for categorical variables.

**Table 4:** Odds ratios (OR) with 95% confidence intervals (CI) for variables associated with lower cognitive function.

Independent Variable		Model I			Model II			Model III	
	OR	95% CI	*p*-Value	OR	95% CI	*p*-Value	OR	95% CI	*p*-Value
Cheese intake, yes	0.322	0.168–0.617	0.001	0.392	0.197–0.777	0.007	0.404	0.198–0.824	0.013
Frequency of cheese intake, daily	0.971	0.506–1.864	0.929	0.796	0.401–1.577	0.512	0.793	0.390–1.610	0.521
Type of cheese, white mold cheese	0.692	0.267–1.791	0.448	0.881	0.333–2.329	0.798	0.906	0.339–2.421	0.843
Age, 1 unit				1.170	1.089–1.256	<0.001	1.150	1.066–1.239	0.000
Usual walking speed, 1 unit				0.171	0.062–0.472	<0.001	0.218	0.075–0.638	0.005
Grip strength, 1 unit				1.023	0.982–1.066	0.281	1.010	0.965–1.057	0.672
Calf circumference, 1 unit				0.823	0.747–0.908	<0.001	0.843	0.761–0.934	0.001
Anemia, yes							1.674	0.534–5.253	0.377
DBP, 1 unit **							1.015	0.992–1.038	0.214
Number of teeth, 1 unit							0.988	0.960–1.016	0.388
Creatinine, 1 unit							1.506	0.828–2.739	0.180
Albumin, 1 unit							0.569	0.203–1.595	0.284
Total cholesterol, 1 unit							1.000	0.992–1.008	0.999
Urinary incontinence, yes							1.726	0.954–3.125	0.071
Milk, intake							1.038	0.580–1.855	0.901
Dietary variety score, 1 unit							0.912	0.800–1.039	0.166

** DBP: diastolic blood pressure.

## Data Availability

The datasets analyzed in this study are not publicly available due to ethical and legal restrictions imposed by the Ethics Committee of the Tokyo Metropolitan Institute of Gerontology but are available from the corresponding author upon reasonable request.
